# Strengths of early physical rehabilitation programs in surgical breast cancer patients: results of a randomized control study

**DOI:** 10.1186/1753-6561-7-S1-O5

**Published:** 2013-01-30

**Authors:** Antonio Testa, C Iannace, L Di Libero, F Caracciolo

**Affiliations:** 1 Victor Babes, University of Medicine and Pharmacy, Timişoara, Romania; 2 San Giuseppe Moscati Hospital, Avellino, Italy

## Background

In the immediate post-operative period, breast cancer patients can face many problems including functional limitation of the shoulder, pain and depression. Aim of this study, comparing 2 groups of patients, one that underwent to a physical rehabilitation program (PRP) and one as a control group, is to evaluate: functional improvements of shoulder mobility, analgesic effect of PRP, improvements and/or worsening of quality of life (QoL).

## Methods

Seventy women planned for axillary dissection were included in this study and randomly assigned to treated (TG) or control group (CG). Patients of the TG, underwent first, to assisted cautious mobilization of hand, wrist and elbow and after drainage removal, to twenty physiotherapy sessions. CG did not underwent to physical rehabilitation treatment. Participants were evaluated before surgery and postoperatively at fifth day, first, sixth and twelfth month. Shoulder mobility was estimated through the use of a Myrin goniometer. VAS scale was used to estimate pain perceived. EORTC QLQ-30 and EORTC QLQ-BR23 1 questionnaires were used to assess QoL. ANCOVA test was utilized to compare outcomes between groups regarding joint mobility and pain perceived using baseline data as covariates. Two tailed t-Tests were used for the statistical analysis within groups. Mann–Whitney non-parametric test was used to analyze QoL outcomes.

## Results

Statistically significant difference (p < 0.01) between the two groups, in flexion and abduction movements, was reported. Within group analysis, from preoperative period to 12th month, showed absence of statistically significant difference in the TG, while at same time proved statistically significant difference (p < 0.05) in the CG regarding flexion, abduction and internal rotation movements. This evidence that TG regained normal function at 1 year after rehabilitation treatment while CG was unable to do so for flexion, abduction and internal rotation movements. Statistically significant differences (p < 0.001) were observed in terms of pain perception starting from the first month. Results analysis showed effectiveness of rehabilitation treatment in improving patient’s quality of life.

## Conclusions

Postoperative early PRP in breast cancer patients surgically treated with axillary dissection improves significantly the glenohumeral joint mobility, permitting the patients to regain normal shoulder function, reduces pain and widely improves QoL. Authors recognise it as a very intensive rehabilitation programme. They still believe, however, that early rehabilitation should be part of the standard treatment in patient with breast cancer following axillary dissection having a key role in patient’s physical and psycho-social recovery.
